# Clinico-Haematologic association and prognostic relevance of NPM1 and FLT3-ITD mutations in acute Myeloid Leukaemia

**DOI:** 10.12669/pjms.35.1.285

**Published:** 2019

**Authors:** Rafia Mahmood, Chaudhry Altaf, Hamid Saeed Malik, Saleem Ahmed Khan

**Affiliations:** 1 Rafia Mahmood, MBBS, FCPS, Department of Haematology, Armed Forces Institute of Pathology, Rawalpindi, Pakistan; 2 Chaudhry Altaf, MBBS, FCPS, Department of Haematology, Armed Forces Institute of Pathology, Rawalpindi, Pakistan; 3 Hamid Saeed Malik, MBBS, FCPS, Department of Haematology, Armed Forces Institute of Pathology, Rawalpindi, Pakistan; 4 Saleem Ahmed Khan, MBBS, MCPS, FCPS, FRCP (Edin), PhD, Department of Haematology, Armed Forces Institute of Pathology, Rawalpindi, Pakistan

**Keywords:** Acute myeloid leukaemia, Complete response (CR), Disease free survival (DFS), FLT-3 ITD, NPM-1

## Abstract

**Background & Objectives:**

Molecular genetic abnormalities have a significant role not only in diagnosis but also in determining the clinical course and prognosis. Nucleophosmin-1 (NPM-1) is associated with good prognosis while internal tandem duplication of the fms-like tyrosine kinase-3 gene (FLT3-ITD) confers a poor prognosis. Knowledge of the status of these mutations in AML patients not only guides treatment decisions but also helps in predicting response to frontline induction and consolidation chemotherapy as well as the risk of relapse and overall survival. Our objectives were to determine the prevalence, clinico-haematological features and immunophenotypic characteristics of AML patients with FLT3-ITD and NPM1 mutation and to evaluate the response to induction therapy (CR) and disease free survival (DFS) in this cohort of patients.

**Methods:**

Patients diagnosed as AML from March 2015 to March 2017 at Armed Forces Institute of Pathology Rawalpindi were included in the study. Clinico-haematologic and immunophenotypic parameters were noted and molecular analysis for FLT3-ITD and NPM1 mutation was performed. Any correlation with cytogenetics or other molecular markers was also studied. Response to standard induction chemotherapy and disease-free survival were assessed.

**Results:**

A total of 108 cases of AML were analyzed. Median age was 35 years and 64.8% were males. The median age of the study group was 35 years. Of these, 70 (64.8%) were males while 38 (35.2%) were females. Twenty-nine (26.9%) patients were NPM1 positive, twelve (11.1%) were FLT3-ITD positive while eight (7.4%) were positive for both mutations. Patients with NPM1 mutations were associated with female gender, higher haemoglobin level and platelet counts while those with FLT3-ITD mutations were predominantly seen in male patients and had significantly higher WBC counts, bone marrow blasts, biopsy cellularity and LDH levels. CR rates of NPM1 positive, FLT3-ITD positive and both mutation positive groups were 72%, 60% and 71%, respectively. The median disease-free survival was significantly lower in the FLT3-ITD positive group (7.1 months) as compared to the NPM1 positive group (16.1 months). The median disease-free survival was 12 months and 11.9 months in the NPM1 positive/FLT3-ITD positive and the NPM1 negative/FLT3-ITD negative groups, respectively.

**Conclusion:**

AML patients harbouring NPM1 and FLT3-ITD mutations have distinct clinical and haematological characteristics. NPM1 mutations have a better CR and DFS as compared to FLT3-ITD group.

## INTRODUCTION

Acute myeloid leukaemia is a heterogenous group of disorders characterized by abnormal proliferation of clonal haemopoietic progenitor cells.^1^ Over the years, better understanding of the biology of disease has led to the identification of cytogenetic aberrations and molecular genetic alterations that not only have a role in leukemogenesis but also in disease progression.^2^ Specific genetic abnormalities define specific AML disease entities, thus being essential for diagnosis while others are known to have a role in determining prognosis, predicting response to treatment and overall survival.^3^ These genetic molecular markers may be therapeutic targets and can help guide treatment decisions. Moreover, these can be used for monitoring of minimal residual disease.^4^

Nucleophosmin, nucleolar phosphoprotein B23, located within the nucleolus, is a nucleocytoplasmic shuttling protein involved in chaperoning ribosomal proteins and core histones from the nucleus to the cytoplasm as well as regulating the ARF-p53 tumor suppressor pathway.^5^ Mutations in the nucleophosmin (NPM1) gene are the most frequently occurring gene mutations in AML.^6^ These mutations identify patients that respond better to chemotherapy and have improved outcomes.^7^

Fms like tyrosine kinase 3 (FLT-3) is involved in normal haematopoiesis regulating differentiation, proliferation and survival of hematopoietic progenitor cells.^8^ Internal tandem duplications of the juxtamembrane domain result in constitutive activation of the FLT-3 receptor tyrosine kinase and in AML are a prognostic indicator associated with adverse disease outcome.^9^

The present study was designed with an aim to see the prevalence of NPM1 and FLT3-ITD mutations in our part of the country. Our aim was to see the clinical relevance and prognostic significance of these mutations as well as the response to treatment in Pakistani AML patients. Knowledge of the status of these mutations in AML patients not only guides treatment decisions but also helps in predicting response to frontline induction and consolidation chemotherapy as well as the risk of relapse and overall survival.

## METHODS

A total of one hundred and eight patients diagnosed as AML over a period of two years (March 2015 to March 2017) were included in the study. They belonged to different ethnic groups. Patients of all age groups and both genders were included. Diagnosis of AML was made on routinely stained peripheral blood smears and bone marrow aspiration/biopsies according to WHO criteria and these patients were also classified according to French-American-British (FAB) criteria.

### Clinico-Haematological Parameters

Detailed history and complete physical examination was done. Symptoms and signs were noted. Diagnostic work-up included complete blood counts (CBC), peripheral blood film, chest X-ray and abdominal ultrasonography. Serum biochemical profile was done in all patients and lactate dehygronase (LDH) levels noted. Bone marrow examination was performed. Bone marrow blast percentage and biopsy cellularity were noted.

### Immunophenotyping

Immunophenotyping was done at the time of diagnosis. One ml of whole blood/bone marrow sample was used for immunophenotyping by flow cytometry and monoclonal antibodies obtained from Becton-Dickinson Biosciences USA. Two color flow cytometry was performed on FACS Flow Cytometer (Becton-Dickinson Biosciences USA).

### Molecular Analysis for NPM1 and FLT3-ITD

Two ml whole blood/bone marrow sample was used for molecular studies. PCR for NPM1 mutation was performed using complimentary DNA by Amplification Refractory Mutation System Methodology. NPM1 was amplified by genomic PCR using specific primers. Amplification was performed in a thermal cycler (Applied Biosystems). The samples were then run on gel electrophoresis using 6% polyacrylamide gel along with positive and negative controls. For FLT3-ITD mutation analysis, conventional PCR using allele specific primers were used to amplify the ITD mutant region. For gene amplification, denaturation at 95°C for five minutes, annealing at 50°C for 45 sec, extension at 72°C for 50 sec, final extension at 72°C for seven minutes and hold at 22°C for 5 min was done. The FLT-3 amplificons were then subjected to gel electrophoresis using 6% polyacrylamide gel. Samples showing additional longer PCR products were considered FLT3-ITD positive. Positive and negative controls were also run.

### Molecular Analysis for Detection of Fusion Genes

Reverse transcription polymerase chain reaction (RT-PCR) was performed to detect fusion genes AML1-ETO, CBFB-MYH11, PML-RARA and BCR-ABL and any association of these fusion genes with NPM1 and FLT3-ITD mutation was studied. RNA was extracted from 2-4ml of blood/ bone marrow sample collected in EDTA. cDNA was synthesized from extracted RNA by reverse transcription. The cDNA product was amplified with Taq polymerase and amplification plots were generated on ABI 7500 Real Time PCR system. Positive samples were identified when fluorescence exceeded threshold limit.

### Cytogenetic Analysis

Conventional cytogenetics was performed using Giemsa trypsin banding technique and interpretation was done according to ICSN criteria. Any correlation with the mutations under study was noted.

### Treatment and Response to Induction Chemotherapy

All patients were given standard “3+7” induction chemotherapy with daunorubicin (45mg/m^2^)and cytarabine (100 mg/m^2^) followed by consolidation therapy with high dose cytosine arabinoside (3g/m^2^ 12hrly on day 1, 3 and 5). Bone marrow examination was done on recovery of counts to evaluate response. Complete response (CR) was defined as ANC ≥ 1.5x10^9^/l, platelets > 100x10^9^/l with less than 5% blasts in a cellular marrow. Disease-free survival was defined as the time in months from complete response to relapse or death.

### Statistical Analysis

Collected data was entered and analyzed using SPSS version 20. Baseline characteristics and prognostic factors were compared by using chi-square test. All p values were two-sided. P≤0.05 was considered significant.

## RESULTS

A total of one hundred and eight patients diagnosed as AML were included in the study. The median age of the study group was 35 years. Of these, 70 (64.8%) were males while 38 (35.2%) were females. The frequency of NPM1 mutation was 34.3% and FLT3-ITD mutations was 18.5%.

On basis of molecular analysis for NPM1 mutation and FLT3-ITD, these patients were divided into four groups:

Group-A - *NPM1 positive FLT3-ITD negative*
Group-B - *NPM1 negative FLT3-ITD positive*
Group-C - *NPM1 positive FsLT3-ITD positive*
Group-D - *NPM1 negative FLT3-ITD negative*

Twenty-nine (26.9%) patients were in Group-A, Twelve (11.1%) were in Group-B, Eight (7.4%) were in Group-C and Fifty-nine (54.6%) were in Group-D. The clinico-haematologic characteristics of these patients are summarized in Table-I.

**Table-I:** Clinico-haematologic characteristics of the different groups

	NPM-1 pos, FLT3-ITD neg n=29	NPM-1 neg, FLT3-ITD pos n=12	NPM-1 pos, FLT3-ITD pos n=8	NPM-1 neg, FLT3-ITD neg n=59
Median age (years)	38	32	33	37
Male gender (%)	55.2	75	62.5	67.8
Median WBC count (x10^9^/l)	34.1	59.2	48.6	39.8
Median Haemoglobin (g/dl)	8.2	7.4	7.9	7.9
Median Platelet count (x10^9^/l)	69	42	59	56
Median LDH level (U/ml)	442	716	584	412
Bone marrow blasts (%)	70	90	85	67
Biopsy cellularity (%)	80	95	90	80

The FLT3-ITD group presented at a relatively earlier age when compared to other groups, though, there was no statistically significant association seen between any of the groups with the age of presentation. Patients showing only NPM1 mutations were significantly associated with female gender, higher haemoglobin levels and platelet counts when compared to other groups. FLT3-ITD mutations were predominantly seen in male patients and this finding was statistically significant. Patients who had FLT3-ITD mutations had significantly higher WBC counts, bone marrow blasts and biopsy cellularity at the time of presentation. FLT3-ITD was also positively correlated with higher LDH levels.

Patients harbouring NPM1 mutations presented with de novo AML while FLT3-ITD mutations were seen in two patients having MDS related AML and one patient having therapy related AML (Table-II). Patients with FLT3-ITD mutations were predominantly found in FAB M1 and M4. Two patients with FLT3-ITD had AML M3 while NPM1 mutations were not found in any patient of AML M3. NPM1 mutations were mostly seen in AML M2 followed by M1, M4 and M5 while M2 and M1 were the typical FAB subtypes in patients having both mutations.

**Table-II:** Distribution in to different FAB types.

	NPM-1 pos, FLT3-ITD neg n=29	NPM-1 neg, FLT3-ITD pos n=12	NPM-1 pos, FLT3-ITD pos n=8	NPM-1 neg, FLT3-ITD neg n=59
De Novo AML	29 (100%)	9 (75%)	8 (100%)	56 (94.9%)
MDS related AML	-	2 (16.7%)	-	3 (5.1%)
Therapy re-lated AML	-	1 (8.3%)	-	-
***FAB type***				
M0	1 (3.4%)	0 (0%)	0 (0%)	5 (8.5%)
M1	6 (17.2%)	5 (4.6%)	3 (37.5%)	10 (16.9%)
M2	14 (13%)	1 (8.3%)	3 (37.5%)	19 (32.2%)
M3	0 (0%)	2 (16.7%)	1 (12.5%)	11 (18.6%)
M4	3 (10.3%)	3 (25%)	1 (12.5%)	9 (15.3%)
M5	3 (10.3%)	0 (0%)	0 (0%)	3 (5.1%)
M6	2 (6.9%)	1 (8.3%)	0 (0%)	2 (3.4%)
M7	0 (0%)	0 (0%)	0 (0%)	0 (0%)

Immunophenotypically, patients with NPM mutations had lower expressions of HLA-DR and CD34 as compared to those having FLT3-ITD mutations. However, no other significant association with any other immunophenotypic marker was seen. On cytogenetic analysis, 82.8% patients in Group-A (*NPM1 positive FLT3-ITD negative*), 75% of Group-B (*NPM1 negative FLT3-ITD positive*) patients, 87.5% of Group-C (*NPM1 positive FLT3-ITD positive*) patients and 78% of Group-D (*NPM1 negative FLT3-ITD negative*) patients had a normal karyotype. We also studied any association of these mutations with any of the molecular markers. Two patients with FLT3-ITD had PML-RARA fusion gene also while no other molecular marker was detected in this subgroup. AML1-ETO translocation was detected in 2 patients having NPM1 mutations. No patient with NPM1 mutation, FLT3-ITD mutation or both had CBFB-MYH11 or BCR-ABL. However, further studies on larger number of patients are needed to establish any statistically significant association.

**Fig.1:**
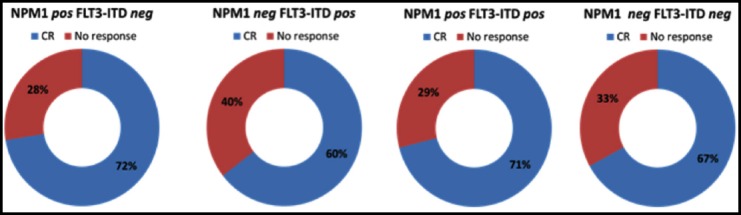
Complete response of the different subgroups.

These patients were given standard induction chemotherapy. Patients with AML M3 were excluded from the study because they were treated with a different regimen. Four patients were lost to follow-up. Fig.1 shows the number of patients that achieved complete response in each subgroup. Patients with NPM1 mutations and those with both NPM1 and FLT3-ITD mutations had comparatively better CR rate as compared to those with only FLT3-ITD mutations or those not having either of these mutations.

**Fig.2:**
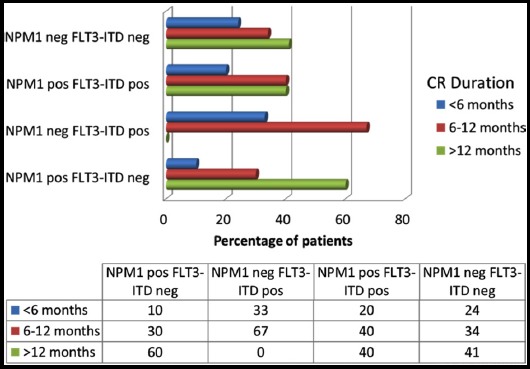
CR duration of the patients.

The patients who achieved a complete response were then followed up and duration of complete response (CR) was noted to see the disease-free survival. Four patients were lost to follow up. Among patients who achieved a CR, the median disease-free survival was significantly lower in the FLT3-ITD positive group (7.1 months) as compared to the NPM1 positive group (16.1 months). The median disease-free survival was 12 months and 11.9 months in the NPM1 positive/FLT3-ITD positive and the NPM1 negative/FLT3-ITD negative groups, respectively. The patients of each group were then classified into 3 categories - those having CR duration of less than 6 months, CR duration 6-12 months and CR duration more than 12 months. FLT3-ITD positive AML patients had a significantly shorter CR duration as compared to the other groups (Fig.2).

## DISCUSSION

Genetic testing incorporating both cytogenetic karyotyping and molecular analysis has an important role in defining and classifying AML patients.^10^ Specific cytogenetic and molecular features defining specific disease entities permit classification of AML patients.^11^ These genetic molecular markers help predict the likelihood of attaining a complete remission and subsequent disease-free survival in patients of acute myeloid leukaemia.^4^ Information regarding the genetic landscape of AML and its impact on prognosis is important in guiding treatment decisions.^1^

NPM1 mutations identify patients with a good prognosis. In the Pakistani population, the frequency of NPM1 mutation was 34.3%. These findings are similar to those reported by Suzuki et al in the Japanese population. Thiede C et al.^12^ has reported NPM1 mutation in 27.47% of his patients. However, Becker H et al.^13^ in a study conducted in Germany has reported a much higher frequency of 56%. FLT3-ITD mutations confer a poor prognosis. In our study the frequency of FLT3-ITD mutation was 18.5%. Elyamany G et al.^14^ have reported a frequency of 14.4% in the Saudi AML patients. FLT3-ITD mutations were found in 19.2% of the AML patients in Thailand by Kumsaen P et al.^15^ However, both the studies by Elmany G et al.^14^ and Kumsaen P et al.^15^ have included both the paediatric and adult population while we have only studied these mutations in adults.

NPM1 was seen in 45% females in our study population. Chauhan PS et al.^16^ has reported NPM1 mutations to be more frequent in females. In our study, NPM1 mutations were associated with higher haemoglobin levels and platelet counts. This is in accordance to the findings reported by Chauhan PS et al.^16^ in the Indian AML patients with NPM1 mutation. Our findings support the observation of Chauhan PS et al^16^ that significantly higher WBC counts are seen in the FLT3-ITD group.

When stratified, patients with NPM1 mutations were seen in FAB type M2 followed by M1, M4 and M5 while Thiede C et al.^12^ has reported NPM1 mutations in FAB M2, M5a and M5b. FLT3-ITD mutations were seen in FAB type M1 and M4 patients in our study. Similar findings have been reported by Thiede C et al.^12^

Following standard induction chemotherapy, the CR rate of NPM1 positive patients was 72%. Our observation matches with that of Chauhan PS et al.^16^ and Schnittger S et al.^17^ who have reported a CR of 73% and 70%, respectively. However, Mullighan CG et al.^18^ and Dohner K et al.^19^ have reported much higher CR rates of 100% and 86%, respectively while Thiede C et al.^12^ has reported a lower CR rate of 58.6%. The CR of patients harbouring the FLT3-ITD mutations was 60% in our population as compared to a much lower CR of 49.3% reported by Theide C et al.^12^

Keeping in view its importance, molecular analysis for NPM1 and FLT3-ITD mutations should be included in the initial workup of all acute myeloid leukaemia patients. NPM1 mutations have been seen to be the most common abnormality with specific presenting features and a good outcome. Considering this, WHO in its 2016 revision has recognized AML with NPM1 mutations as a discrete entity and has classified it in the category of AML with recurrent genetic abnormalities. FLT3-ITD mutations remain an important prognostic marker in AML defining disease with a higher probability of relapse and a poorer overall survival. Patients with this mutation may be candidates for more specific targeted therapy like FLT3 inhibitors. Also, treatment decisions regarding consolidation may be altered bearing in mind the adverse clinical impact of FLT3-ITD in AML.

In developing countries like Pakistan with few tertiary care centres having facilities for molecular analysis, we at Armed Forces Institute of Pathology cater to a large number of patients from different parts of the country. We have conducted this study to establish the prevalence of these mutations in our population and to evaluate the association of these mutations with clinico-haematologic parameters, immunophenotypic markers, cytogenetic and molecular alterations. Molecular analysis for NPM1 and FLT3-ITD mutations must be performed to risk stratify our patients, determine treatment protocols and predict prognosis.

## References

[1] Burnett AK, Grimwade D, A Victor Hoffbrand, Douglas R Higgs, David M Keeling, Atul B Mehta (2016). Acute Myeloid Leukaemia. Postgraduate Haematology.

[2] Mrozek K, Marcucci G, Nicolet D, Maharry KS, Becker H, Whitman SP (2012). Prognostic significance of the European Leukemia Net standardized system for reporting cytogenetic and molecular alterations in adults with acute myeloid leukemia. J Clin Oncol.

[3] Papaemmanuil E, Gerstung M, Bullinger L, Gaidzik VI, Paschka P, Roberts ND (2016). Genomic Classification and Prognosis in Acute Myeloid Leukemia. N Engl J Med.

[4] Ivey A, Hills RK, Simpson MA, Grech GA, Patel GY, Bhudia N, UK National Cancer Research Institute AML Working Group (2016). Assessment of minimal residual disease in standard-risk AML. N Engl J Med.

[5] Falini B, Martelli MP, Bolli N, Sportoletti P, Liso A, Tiacci E (2011). Acute myeloid leukemia with mutated nucleophosmin (NPM1):is it a distinct entity?. Blood.

[6] Schnittger S, Bacher U, Kern W, Alpermann T, Haferlach C, Haferlach T (2011). Prognostic impact of FLT3-ITD load in NPM1 mutated acute myeloid leukemia. Leukemia.

[7] Liu Y, He P, Liu F, Shi L, Zhu H, Zhao J (2014). Prognostic significance of NPM1 mutations in acute myeloid leukemia:A meta-analysis. Mol Clin Oncol.

[8] Janke H, Pastore F, Schumacher D, Herold T, Hopfner KP, Schneider S (2014). Activating FLT3 mutants show distinct gain-of-function phenotypes in vitro and a characteristic signaling pathway profile associated with prognosis in acute myeloid leukemia. PLoS One.

[9] El Fakih R, Rasheed W, Hawsawi Y, Alsermani M, Hassanein M (2018). Targeting FLT3 Mutations in Acute Myeloid Leukemia. Cells.

[10] Yohe S (2015). Molecular Genetic Markers in Acute Myeloid Leukemia. J Clin Med.

[11] Dohner H, Estey E, Grimwade D, Amadori S, Appelbaum FR, Buchner T (2017). Diagnosis and management of AML in adults:2017 ELN recommendations from an international expert panel. Blood.

[12] Thiede C, Koch S, Creutzig E, Steudel C, Illmer T, Schaich M (2006). Prevalence and prognostic impact of NPM1 mutations in 1485 adult patients with acute myeloid leukemia (AML). Blood.

[13] Becker H, Marcucci G, Maharry K, Radmacher MD, Mrózek K, Margeson D (2010). Favorable prognostic impact of NPM1 mutations in older patients with cytogenetically normal de novo acute myeloid leukemia and associated gene- and microRNA-expression signatures:a Cancer and Leukemia Group B study. J Clin Oncol.

[14] Elyamany G, Awad M, Fadalla K, Albalawi M, Shahrani MA, Abdulaaly AA (2014). Frequency and Prognostic Relevance of FLT3 Mutations in Saudi Acute Myeloid Leukemia Patients. Adv Hematol.

[15] Kumsaen P, Fucharoen G, Sirijerachai C, Chainansamit SO, Wisanuyothin N, Kuwatjanakul P (2016). FLT3-ITD Mutations in Acute Myeloid Leukemia Patients in Northeast Thailand. Asian Pac J Cancer Prev.

[16] Chauhan PS, Ihsan R, Singh LC, Gupta DK, Mittal V, Kapur S (2013). Mutation of NPM1 and FLT3 Genes in Acute Myeloid Leukemia and Their Association with Clinical and Immunophenotypic Features. Dis Markers.

[17] Schnittger S, Bacher U, Kern W, Alpermann T, Haferlach C, Haferlach T (2011). Prognostic impact of FLT3-ITD load in NPM1 mutated acute myeloid leukemia. Leukemia.

[18] Mullighan CG, Kennedy A, Zhou X, Radtke I, Phillips LA, Shurtleff SA (2007). Pediatric acute myeloid leukemia with NPM1 mutations is characterized by a gene expression profile with dysregulated HOX gene expression distinct from MLL-rearranged leukemias. Leukemia.

[19] Dohner K, Schlenk RF, Habdank M, Scholl C, Rücker FG, Corbacioglu A (2005). Mutantnucleophosmin (NPM1) predicts favorable prognosis in younger adults with acute myeloid leukemia and normal cytogenetics:Interaction with other gene mutations. Blood.

